# Multidisciplinary management of breast cancer

**DOI:** 10.1186/s13690-016-0163-7

**Published:** 2016-12-05

**Authors:** Anne-France Leclerc, Guy Jerusalem, Martine Devos, Jean-Michel Crielaard, Didier Maquet

**Affiliations:** 1 Department of Sports and Rehabilitation Sciences, Liège University, Allée des Sports 4 - B21, 4000 Liège, Belgium; 2 Department of Physical Medicine, Liège University Hospital, Avenue de l’Hôpital 1 - CHU B35, 4000 Liège, Belgium; 3 Division of Medical Oncology, Liège University Hospital, Liège University, Avenue de l’Hôpital 1 - CHU B35, 4000 Liège, Belgium; 4 Department of Clinical Hematology, Liège University Hospital, Avenue de l’Hôpital 1 - CHU B35, 4000 Liège, Belgium

**Keywords:** Breast cancer, Public health issue, Side effects, Multidisciplinary supportive care

## Abstract

Breast cancer, with an increasing incidence, is the most frequently diagnosed cancer in women worldwide. The treatments proposed, generally a combination of surgery, radiotherapy, chemotherapy, endocrine therapy and/or targeted therapy, are constantly improving, allowing a reduction in the mortality rate, but they are still causing many side effects, not only early but also late, which leads us to consider the post-cancer period as a chronic condition. Side effects, reviewed in this commentary, may affect physical functions, psychological status, social situation, body composition, well-being and quality of life of the patient. In view of the extent of these areas in which side effects of breast cancer and of its treatments can be found, the supportive care offered at the end of treatment need to be multidisciplinary. Different supportive care interventions may be proposed to the patients such as psychological and behavioral interventions, complementary therapies, diet interventions, physical activity/rehabilitation or also physiotherapy interventions for example, all having shown some beneficial effects in the literature. The benefits of these supportive care interventions are thereby already established and they are described in this article, but others studies will be needed to clearly define indications and most optimal modalities of application to reduce side effects and improve quality of life of patients.

## Background

Because of its incidence, the incurred treatments and especially its multiple clinical and social consequences, breast cancer constitutes a real public health issue.

This cancer remains the most frequently diagnosed in women worldwide with, among others, 1 380 000 new cases reported in 2008 (23 % of all cancers) and its incidence continues to rise [1, 2].

Different treatments may be proposed, generally a combination of surgery, radiotherapy, chemotherapy, endocrine therapy and/or targeted therapy. Their effectiveness is constantly increasing, allowing a reduction in mortality [3, 4]. Thereby, between 1992 and 2002 for example, the death rate from breast cancer has decreased by 13,4 % in Europe for women of all ages and by 16,7 % for those aged 35 to 64 [5].

However, despite this progress, breast cancer and its treatments are still causing many psychological, physical, and social side effects, not only early but also late, which leads us to consider the post-cancer period as a chronic condition. In view of the extent of the areas in which side effects of breast cancer and of its treatments can be found, the supportive care offered during and at the end of treatment need to be multidisciplinary.

We will thus, in this paper, review the various treatment-related side effects and the different supportive care interventions aiming to improve them.

## Main text

### Side effects of breast cancer and of its treatments

#### Surgery

Surgery, including radical mastectomy and lumpectomy, associated or not with axillary dissection, remains the centerpiece of breast cancer treatments [6]. Although the techniques continue to improve, various side effects can be observed. The most common complication encountered directly after breast surgery is wound infection, with an incidence of 4,34 % 30 days after radical mastectomy and 1,97 % after lumpectomy with axillary dissection [7]. Cardiac and pulmonary complications are uncommon with a rate of 0,12 % after mastectomy and 0,66 % after lumpectomy and axillary dissection [7]. Similarly, central nervous system complications are rare with rates of 0,12 and 0,07 % respectively and the urinary tract infection rates are 0,66 and 0,14 %, respectively [7]. Then, surgery remaining a mutilating action in the eyes of patients [6], the image of themselves may be impaired and particularly after radical mastectomy [8, 9]. Similarly, sexual functioning [8, 9] and role in society [9] also appear sometimes altered. Finally, surgery is also found responsible for fatigue and pain [10].

Axillary dissection can cause additional side effects including lymphedema, numbness, loss of strength and reduction of arm movement amplitude [8, 10–12] reducing the quality of life [8, 12]. The incidence rate published for lymphedema varies between 2 % and 65 % depending on the surgical technique, the axillary sampling method and the use of chemotherapy and radiotherapy [13].

#### Radiotherapy

Short-term side effects of radiation therapy are erythema observed in over 50 % of women [14–16], the dry peeling in 6 to 10 % of cases, or wet in more than 50 % of cases [10, 14–16], fatigue [10, 15, 16] more or less pronounced depending on the treatment history and present in over 50 % of women [14], but also edema of the subcutaneous fat [10, 16], esophagitis following irradiation of the mammary chain [16], hyperpigmentation [10, 15, 16], myelosuppression [14], pain [10, 15, 17] as well as anxiety, depression and decreased quality of life [14].

The long-term effects, however rare, include lymphedema of the arm [10, 12, 16] present in 6 to 10 % of patients after lymph node irradiation [14], subcutaneous fibrosis with possible telangiectasia [16, 17] in 10 to 50 % of cases [14], the pulmonary disorders including pneumonia with fever, cough and breathlessness [10, 16] with an incidence of less than 1 % [14], brachial plexitis [10, 15–17] as well as heart problems such as arrhythmias, pericarditis, ischemic heart disease and myocardial infarction [10, 16, 17] again present in less than 1 % of cases [14].

#### Chemotherapy

Chemotherapy is an important systemic treatment in the management of breast cancer. Depending on the drugs administered to the patient, it can cause various side effects including asthenia [10, 14, 18–22] which is the most common. Asthenia is, indeed, encountered in 70 to 100 % of patients during the course of chemotherapy [21, 23], but also thereafter [18, 19, 21]. The intensity of fatigue appears stable during treatment [19, 21] and 60 % of patients present a moderate to severe level of fatigue [23].

Alopecia is another side effect often encountered in the case of chemotherapy [14, 17, 18, 21, 24] and altering the self-image of women [22]. Nausea and vomiting [14, 18, 21, 22, 24], diarrhea [14, 18, 22], leucopenia and neutropenia within days of each cycle [10, 14, 24, 25], infection [18, 25] and the appearance of neurological toxicity with motor and sensory peripheral neuropathies mainly caused by taxanes [10, 14, 24, 25] are other side effects experienced by the patients. In addition, weight gain [10, 14, 18, 21, 24] and ovarian failure resulting in menopausal symptoms including vaginal dryness, hot flashes, dyspareunia, sleep disorders and/or osteoporosis are other frequently observed side effects [10, 14, 18, 20, 24, 26]. Similarly, pain [10, 14, 18, 21], heart problems [10, 14], pulmonary problems [10], cognitive dysfunction with difficulties in memory, concentration and language [10, 14, 20, 24], anxiety and finally depression [21, 27] are other complications of chemotherapy that adversely affect the quality of life of patients [8, 14, 22, 25, 27].

#### Endocrine therapy

Like other treatments, endocrine therapy may cause side effects consisting mainly in menopausal symptoms: vaginal dryness, hot flashes, dyspareunia, sleep disorders or decreased bone density and increased risk of bone fracture [10, 14, 17, 26]. Endocrine therapy may also cause pain, fatigue, an increase in body weight and vasomotor symptoms [10, 14]. Other gynecological and sexual problems are also observed: vaginal discharge, mucosal atrophy, loss of libido and dyspareunia [10, 28].

#### Targeted therapy

Finally, targeted therapy or immunotherapy is indicated in the breast cancer treatment if there is an overexpression of the HER2 protein (15 to 20 % of cases) and/or an amplification of the gene [29, 30]. Although the therapy is thus more selective, it unfortunately also generates adverse effects. Depending on the drug used, nausea [31, 32], diarrhea [31, 32], fatigue [32], headache [32], fever [29, 30, 32], cardiac side effects [29, 30, 32] and dermatological disorders [31, 32] may appear.

In summary, therapy of breast cancer and outcome is improving but side effects are still frequent and are best managed by a multidisciplinary team.

### Supportive care

Supportive care aim to improve the quality of life of the patient by different interventions including symptom control, psychological and social supports, physical rehabilitation, educational needs and sometimes the end of life care, which requires a multidisciplinary cooperation and coordination [33–35].

Thus, to meet the patient's needs, we will discuss different supportive care interventions that can provide help and support and which have already been evaluated in several clinical.

#### Psychosocial and behavioral interventions

Different systematic reviews and meta-analyses have already analyzed the benefits provided by psychosocial and behavioral interventions to patients who have been treated for breast cancer [36–42]. These interventions include the terms “individual, group or couple psychotherapy, psychosocial therapy, psychoeducation, emotional and social support group, cognitive behavioral therapy, health education and telephone or oral counseling and support”. Although they vary in terms of content, duration, frequency and modality of access for the patient, these interventions have a positive impact on emotional or psychological distress [36, 39, 40, 42], anxiety [36, 37, 39], depression [36, 37, 39, 41], fatigue [37], sleep disorders [43, 44], body-image [37], stress [37, 39], relationship functioning [40] and on quality of life [36, 37, 39–41].

#### Complementary therapies

In complementary therapies, we include relaxation, meditation, yoga, music therapy, stress management and massage. These interventions may also be recommended as supportive care options during and/or after breast cancer treatment because they also show positive effects on anxiety [36–38], depression [36–38], emotional distress [36], mood disorders [38], stress [37, 38], fatigue [37, 38], sleep disruption [38, 44] and quality of life [36, 38]. However, as psychosocial and behavioral interventions, these interventions should be recommended based on professional judgment and patient preferences.

#### Diet intervention

Overweight or obesity at the time of diagnosis is a poor prognosis factor and may be associated with a variety of undesirable outcomes such as an increase in the risk of recurrence and disease-specific or overall mortality [45–48]. Moreover, weight gain is a common side effect after chemotherapy [10, 14, 18, 21, 24] and/or endocrine therapy [10, 14] and it seems to be the result of an increase in adipose tissue mass [45]. To prevent this weight gain and to improve food choices, the American Cancer Society wrote a report in which they discuss nutrition guidelines for cancer survivors [45]. According to this guideline, breast cancer survivors should try to maintain a healthy weight by eating a balanced diet and by practicing regular physical activity. Diets should emphasize vegetables and fruits, have low amounts of saturated fats and include sufficient dietary fiber [45, 47]. Furthermore, other studies show the efficacy of in-person and telephone counseling/nutrition education in weight loss strategies and dietary improvement in women treated for breast cancer [48, 49].

#### Physical activity/rehabilitation

To prevent weight gain, but also for its many beneficial effects on psychological and physical aspects, a regular physical activity is recommended for patients treated for breast cancer [10, 37, 50, 51]. Inactivity should be avoided and daily activities should be resumed as soon as possible after surgery and during adjuvant cancer treatment [10, 45, 51]. More specifically, concerning aerobic training, 20 to 60 min of exercise at a moderate intensity (50–75 % of the maximal heart rate) are recommended 3 to 5 times per week [10, 45, 51, 52]. For resistance training, it is recommended to follow a supervised program of approximately 2 to 3 sets of 8 to 15 repetitions with a very low load at baseline, 2 to 3 times a week. The resistance can be increased in small increments without any upper limit, but it is important to monitor the appearance of symptoms of the arm and/or shoulder such as a lymphedema and to reduce the resistance or stop the exercises according to the complications observed [10, 45, 52]. Finally, with regard to stretching, it is advisable to stretch the large muscle groups for 10 to 30 s 2 to 3 times per week [10, 45].

Many clinical trials and systematic reviews have evaluated the benefits of physical training in patients being treated for breast cancer. Although these studies differ according to timing of the intervention (during or after treatment), location (home or institution), duration, frequency and type of exercises performed and thus although no consensus is yet clear on these parameters, many benefits have been objectified. Thus, in view of the literature, physical activity/rehabilitation can have a positive impact on the quality of life of breast cancer patients [10, 37, 50, 53–65], on fatigue [10, 37, 50, 54, 56, 57, 60, 63, 66–68], on cardiopulmonary function (resting and/or maximal heart rate and maximal oxygen consumption) [10, 50, 53, 57, 58, 60, 61, 67–74], on weight and body composition (body weight, body mass index and/or percentage of body fat or lean mass) [10, 50, 57, 61, 66, 69, 71, 75–77] and on the psychological, emotional and physical well-being (such as anxiety, depression, body-image) [10, 37, 50, 54, 55, 57, 60, 61, 67, 72, 75].

#### Physiotherapy interventions

Lymphedema is a side effect often encountered as a result of breast cancer surgery and/or radiotherapy. The physiotherapist, with a manual lymphatic drainage associated with the implementation of compression bandages and/or compression therapy, skin care and remedial exercises, may help to reduce swelling, but also pain and heaviness [12, 78]. Moreover, early physiotherapy interventions (including manual lymphatic drainage, massage of scar tissue, shoulder exercises, and educational strategy) also seem to be effective in the prevention of lymphedema [78].

Finally, all these interventions have shown in the literature the benefits they could bring as supportive care, but all should not always be prescribed to all patients. These supportive care modalities should be administered by qualified and experienced providers which take into account the risk-benefit ratio for each therapy, the patients’ clinical characteristics (such as stage of disease, the overall goal of anticancer therapy, patient performance status and patient adherence), the communication with all health-care providers involved in the patient’s care and also the patient preference [38].

## Conclusions

Due to its high incidence and the side effects experienced as a result of the disease and its treatment, breast cancer constitutes a real public health problem and can be considered as a chronic condition. As side effects affect physical functions and the psychological status, social situation, body composition and well-being of the patient, a multidisciplinary and individualized supportive care is needed in addition to the anticancer therapy. The benefits of various supportive care interventions are already established, but studies are still needed to clearly determine the best modalities of application and the indications based on patient characteristics.
